# Comparative transcriptomic analysis of mice liver treated with different AMPK activators in a mice model of atherosclerosis

**DOI:** 10.18632/oncotarget.15027

**Published:** 2017-02-02

**Authors:** Ang Ma, Dongmei Wang, Yuanyuan An, Wei Fang, Haibo Zhu

**Affiliations:** ^1^ State Key Laboratory for Bioactive Substances and Functions of Natural Medicines, Beijing Key Laboratory of New Drug Mechanisms and Pharmacological Evaluation Study, Institute of Materia Medica, Chinese Academy of Medical Sciences and Peking Union Medical College, China; ^2^ Department of Basic Medical Sciences, Medical College, Xiamen University, Xiamen, China; ^3^ Department of Nuclear Medicine, Fu Wai Hospital, National Center for Cardiovascular Diseases, Chinese Academy of Medical Sciences and Peking Union Medical College, China

**Keywords:** AMPK activators, atherosclerosis, IMM-H007, A-769662, metformin

## Abstract

Atherosclerosis is known to be the primary underlying factor responsible for the development of cardiovascular diseases. Suppression of AMP-activated protein kinase stimulates arterial deposition of excess lipids, resulting in the development of atherosclerotic lesions. In this study we successfully developed the disease model of mice and mimicked the therapeutic effect, for that we chose three different AMP-activated protein kinase activators (IMM-H007, A-769662 and Metformin) to identify which one has a superior effect in the atherosclerosis model. We combined the transcriptomes of four groups of mice liver including high-fat diet group and the experimental groups treated with different AMP-activated protein kinase activators. We analyzed the increased genes to candidate metabolic and disease pathways. Compared to the high-fat diet group, a total of 799 differentially expressed genes were identified in treatment groups. There were 291, 473, and 323 differentially expressed genes in H007, Metformin, and A-769662 group respectively. And seven statistically significant pathways were observed in both H007 and Metformin groups. We expect that gene expression profiling in the mice model would extend our understanding of atherosclerosis in the molecular level. This study provides a fundamental framework for future clinical research on human atherosclerosis and new clues for developing novel drugs for the treatment of atherosclerosis.

## INTRODUCTION

Cardiovascular diseases are the leading cause of morbidity and mortality worldwide and atherosclerosis is known to be the primary underlying factor responsible for the development of these diseases [1]. Individuals with comorbidities such as diabetes and dyslipidemia are prone to develop pathological changes in the arterial wall, in such individuals there are chances that may lead to a state of chronic low grade inflammation and may promote obesity-linked metabolic disorders and cardiovascular disease such as insulin resistance, metabolic syndrome and atherosclerosis [2]. Atherosclerosis is a complex inflammatory disease characterized by the accumulation of lipid rich plaques in arterial walls, which is mediated largely by recruitment of macrophages to the site of injury. It has been reported that genetic variation of apo-E is linked to plasma lipid levels, which is an independent risk determinant of atherosclerosis and coronary heart disease [3]. Atherosclerosis is also a major cause of ischemic stroke. Although carotid artery plaques and cardiogenic emboli have been observed as the embolic sources in patients with cerebral infarction, severe aortic arch atherosclerotic (AAA) plaques may be detected in ischemic stroke patients for whom no embolic source has been identified [4].

AMPK is activated in response to a variety of conditions that deplete cellular energy levels, such as nutrient starvation (especially glucose), hypoxia and exposure to toxins that inhibits the mitochondrial respiratory chain complex-1, 2. AMPK is a serine/threonine protein kinase complex consisting of a catalytic α-subunit (α1 and α2), a scaffolding β-subunit (β1 and β2) and a regulatory γ-subunit (γ1, γ2 and γ3) [5]. AMPK has a key role in maintaining the balance between anabolic and catabolic programs for cellular homeostasis in response to metabolic stress. Given the functional attributes of AMPK in glucose/lipid homeostasis, body weight, food intake, insulin signaling and mitochondrial biogenesis, AMPK is considered to be a major therapeutic target for the treatment of metabolic diseases including type 2 diabetes and obesity [6], hence atherosclerosis. Atherosclerosis is a chronic immune-mediated inflammatory disease of the arterial vessel wall characterized by the thickening of intima primarily due to monocyte recruitment into the sub endothelial space [7]. AMPK activators can effectively inhibit PMA-induced monocyte-to-macrophage differentiation. Also Vascular suppression of AMPK stimulates arterial deposition of excess lipids, resulting in the development of atherosclerotic lesions [8].

In this present study, we chose three different AMPK activators (IMM-H007, A-769662 and Metformin). IMM-H007 (triacetyl-3-hydroxyphenyladenosine) is a derivative of cordycepin, which is an adenosine analog [9]. IMM-H007 was observed to stimulate the phosphorylation of AMPK and to decrease lipid biosynthesis [10]. It has been observed to increase the circulating HDL level, and the cholesterol efflux capacity of HDL, and it can also enhance *in vivo* reverse cholesterol transport (RCT) from macrophages to the plasma, liver, and feces. Furthermore, recent studies showed that ABCA1 suppression by IMM-H007 can reduce atherosclerotic plaque formation in apoE^–/–^ mice [11]. A-769662 is another new activator of AMPK which increase AMPK activity directly through the β1 subunit drug binding site [12]. Metformin is an anti-diabetic drug which activates AMPK indirectly. It affects lipid metabolism, lowering plasma triglycerides and free fatty acids. It was also found to activate the AMPK in intact cells and *in vivo* [13]. Based on the previous reports and studies, we adjusted the dosages of the three AMPK activators. And he three AMPK agonist lead to the similar comparable effects by different mechanisms.

There are many advantages of using mice for experimental atherosclerosis research. For their generation time is only about 9 weeks [14]. A chronological analysis of atherosclerosis in the apoE deficient mouse has shown that the sequential events involved in lesion formation are strikingly similar to those in well-established larger animal models of atherosclerosis and in humans [15].

In this study we first established mice models of atherosclerosis, which were fed with high fat-diet and combined with the above mentioned AMPK activators separately. The purpose of this study was to identify which AMPK activator has a superior effect in the treatment of atherosclerosis. Since liver is the major organ for drug and lipid metabolism, systematic studies of gene expression in hepatic cells treated with high-fat diet and drugs will provide abundant biomarkers for understanding the basic molecular mechanism of drug metabolism and protection against atherosclerosis using AMPK activators. In recent years, global gene expression analysis approaches based on microarray and RNA sequencing has been widely applied in various biomedical studies. For analysis of these transcriptome data (especially in cancers and cardiovascular diseases), biological pathways or networks contributes an important role for connecting different genes and understanding the molecular mechanisms of various pathophysiological processes [16, 17]. Thus, based on high-throughput sequencing technology, we constructed and combined the transcriptomes of four groups of mice liver including high-fat diet group (the control group) and three experimental groups treated with different AMPK activators. Then, we mapped the increased genes to candidate metabolic and disease pathways and systematically compared the differences of these gene between different experimental groups.

Gene-expression profiling of atherosclerosis has recently been used to identify genes and pathways relevant to vascular pathophysiology. This study will help us to broaden our knowledge in the molecular mechanism of drug metabolism and protection against atherosclerosis using different AMPK activators, and this will also provide new clues for developing novel drugs for the treatment of atherosclerosis.

## RESULTS

### Construction of mice models treated with high-fat diet and three AMPK activators

**Figure 1:**
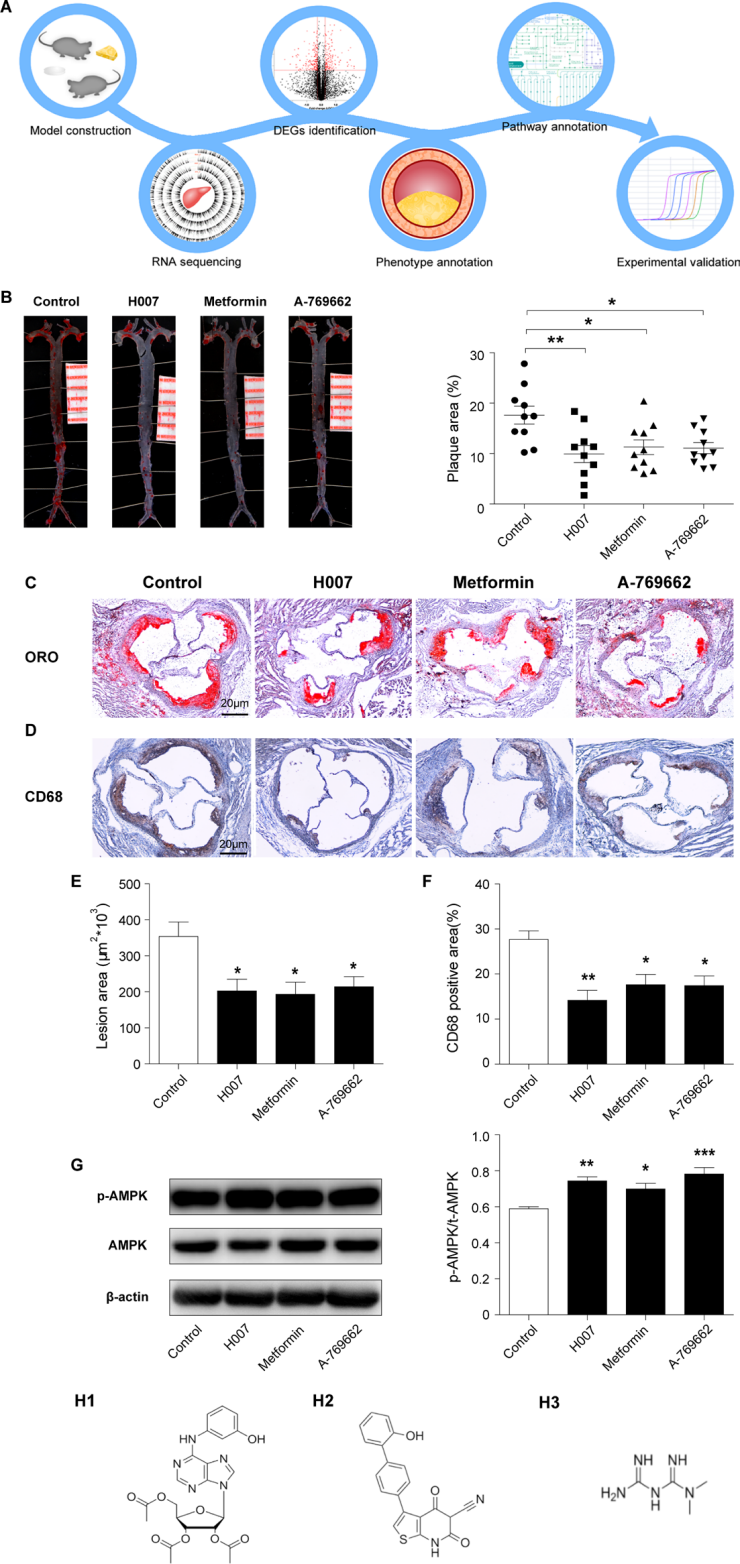
H007 alleviates atherosclerotic plaque development in apoE^–/–^ mice (**A**) Flow chart representing the experimental design and model construction. (**B**) Representative image of en face whole aortas from apoE^–/–^ mice (Scale bars: 1.0 cm) and Graphical representation of the percent of aortic lesion area (lesion area compared to total arch area). (**C**) Representative image of Cryosections of mice aortic roots from control and drugs-treated apoE^–/–^ mice (Scale bars: 20 μm). (**D**) Immunohistochemical staining for the macrophage marker CD68 (Scale bars: 20 μm). (**E**) Graphical representation of lesion area stained by Oil Red O in the aortic root from control and drugs-treated apoE^–/–^ mice. (**F**) Graphical representation of the percent of CD68 positive area compared to total aortic root area determined by ImageJ software. (**G**) Representative image of the protein expression of pT172-AMPK, AMPK and β-actin in liver tissues from apoE^–/–^ mice and Graphical representation of the percent of Thr-172 phosphorylation of AMPK (pT172-AMPK compared to total-AMPK). (**H**1) Structure of IMM-H007; (**H2**) Structure of A-769662; (**H3**) Structure of Metformin.

A flow chart representing the experimental design and model construction was shown in (Figure 1A) at first mice were fed with high fat diet and 3 different AMPK activators separately, after the 10 weeks treatment, mice were killed and their aorta were obtained, then RNA sequencing was done, further identified DEGs (differentially expressed genes) between experimental and control groups (Figure 1B) the whole aorta was obtained for staining, where we found maximum lesion area for the control group. (Figure 1C, 1D) cryosections of aorta containing plaques stained with Oil red O and CD68 respectively. (Figure 1E) Graphical representation of Lesion area in the aortic root from control and drugs-treated apoE^–/–^ mice, with maximum of 353.8682 (units) for control group and 202.6138 (units), 193.4254 (units) and 214.2085 (units) for IMM-H007, Metformin and A-769662 respectively revealed reduction in the lesion area. (Figure 1F) Graphical representation of the percentage of CD68 positive area compared to total aortic root area in cryosection determined by software ImageJ analysis found that, 27.7% positive area for the control group and 14.2%, 17.6% and17.5% for IMM-H007, Metformin and A-769662 respectively relieved reduction in positive area. (Figure 1G) The protein expression of pT172-AMPK, AMPK and β-actin in liver tissues from apoE^–/–^ mice were measured by Western blot. The Thr-172 phosphorylation of AMPK, which reflects AMPK activity, was increased by 26% in the IMM-H007 group, by 19% in the Metformin group, and by 33% in the A-769662 group, versus the control group. And the structures of these three drugs were shown in Figure 1H.

### Global gene expression analysis of mouse liver by RNA-seq

**Figure 2:**
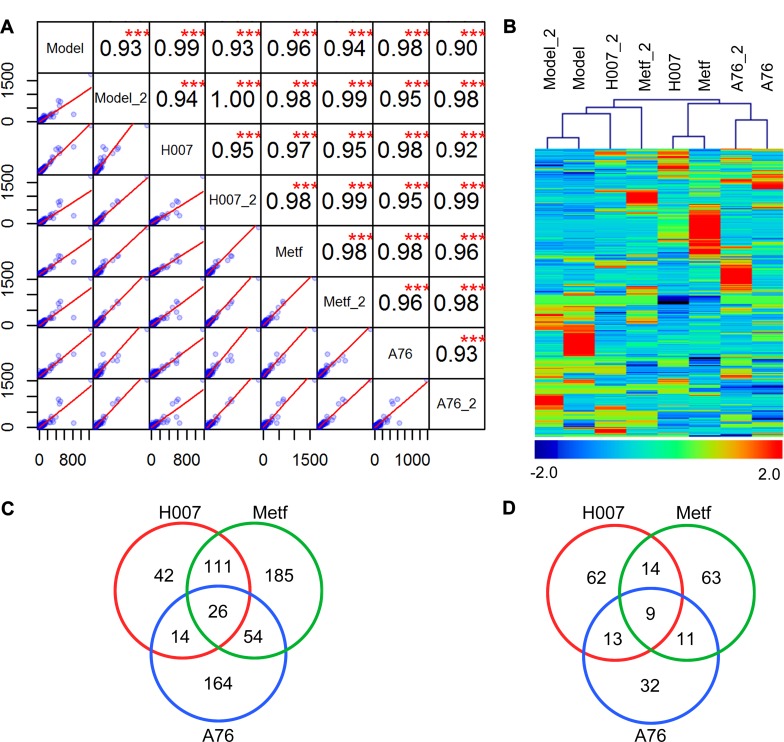
Summary of RNA-seq analysis (**A**) Correlation analysis between replications and experimental groups. (**B**) Cluster analysis of genes and samples using DEGs. (**C**) Comparison of up-regulated DEGs between different groups. (**D**) Comparison of down-regulated DEGs between different groups.

Combining the results of two experimental replications, a total of 20047 unique genes were identified in mouse liver treated with different AMPK activators (Supplementary Information 1). The correlation analysis showed that the overall expression levels are similar between two replications or different groups, which also indicated the high quality of RNA-seq analysis to some extend (Figure 2A). Compared to the control group (Model), a total of 799 DEGs were identified in treatment groups (Supplementary Information 2). There are 291, 473, and 323 DEGs in H007, Metf, and A-76 group respectively. Cluster analysis of DEGs showed that all three treatment groups displayed distinct expression patterns compared to Model group (Figure 2B). However, we also observed that the expression levels of many DEGs were different between replications (within the same group), which indicated that obvious biological variations were existed in mice liver. We further compared the composition of DEGs between three treatment groups (Figure 2C–2D). We observed that all treatment groups have more up-regulated genes than down-regulated genes. In addition, we noted that H007 and Metf shared many DEGs (including 137 up-regulated genes and 23 down regulated genes), which could partially explain that these groups are not clustered within replications.

**Figure 3:**
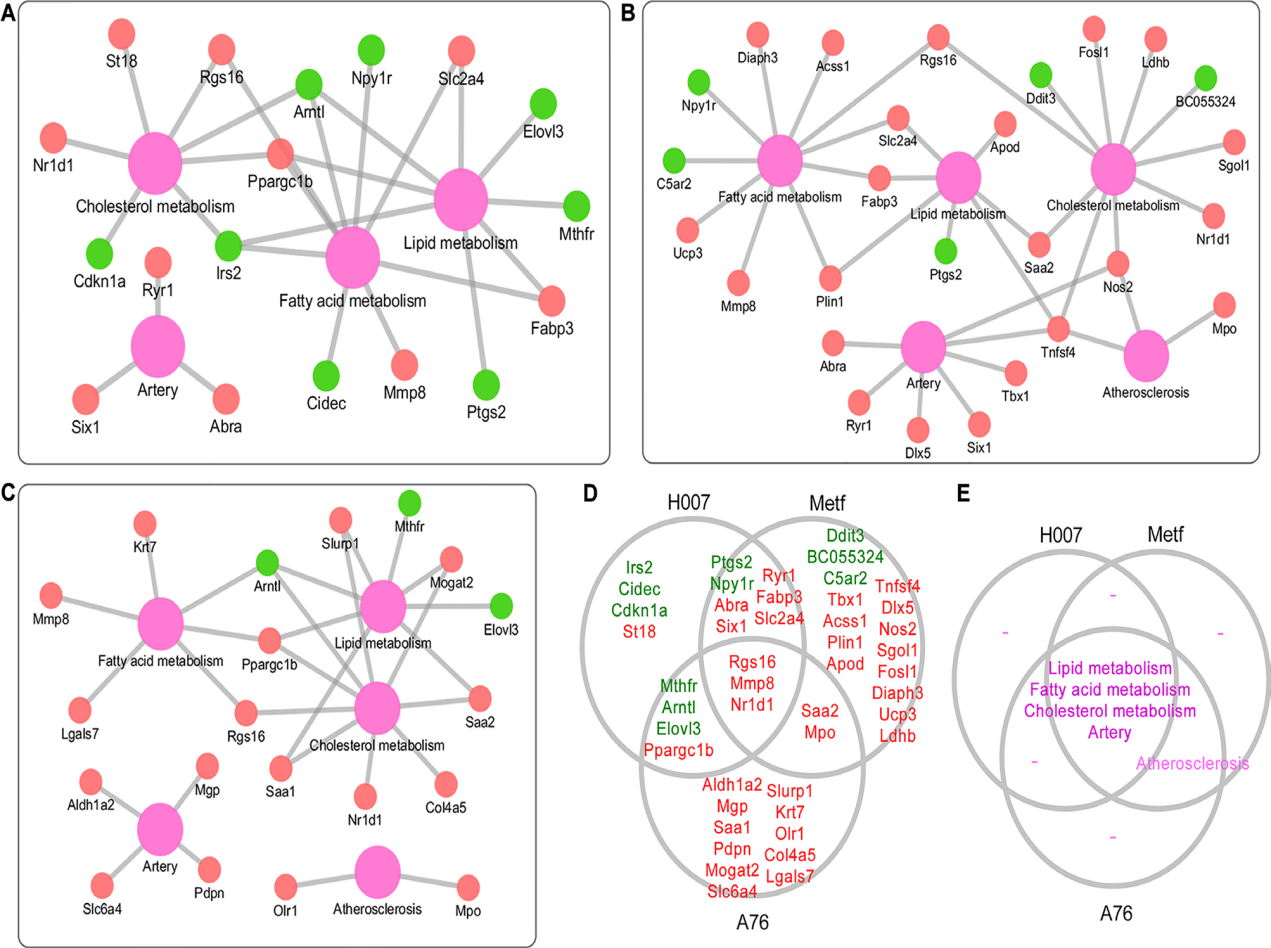
Annotation of DEGs with known phenotypes (**A**) Gene-phenotype network for H007 group. (**B**) Gene-phenotype network for Metf group. (**C**) Gene-phenotype network for A-76 group. (**D**) Comparison of DEGs with known phenotypes between different groups. (**E**) Comparison of phenotypes between different groups. Red indicates up-regulation, while green represents down-regulation. Purple represents phenotype.

Experimental evidences showed that all the three AMPK activators can rescue the pathological changes of atherosclerosis (Figure 1B–1F). Thus, RNA-seq analysis was applied to discover molecular biomarkers that may contribute to this treatment effect. Then we evaluated the feasibility of RNA-seq analysis for identifying bonafide biomarkers using known genes that associated with atherosclerosis. We systematically annotated DEGs based on MGI phenotype annotation. We focused on five types of phenotypes including atherosclerosis, artery, cholesterol metabolism, fatty acid metabolism, and lipid metabolism. A total of 46 DEGs were mapped to 49 subclasses of phenotypes (Supplementary Information 3). We constructed the gene-phenotype relation networks for the three treatment groups separately (Figure 3A–3C). Consistent with the overall number of DEGs, H007 and Metf shared more genes than any other group pairs (Figure 3D, including 8 up-regulated and 2 down regulated). All three groups have DEGs annotated with artery, cholesterol metabolism, fatty acid metabolism, and lipid metabolism phenotypes (Figure 3E). However, only Metf and A-76 group identified DEGs directly associated with atherosclerosis (only two gene were involved, including Tnfsf4 and Olr1). Actually, these DEGs were identified from liver tissue, thus it is reasonable that DEGs are more directly associated with liver metabolism rather than artery and atherosclerosis. Nevertheless, the present RNA-seq analysis did identified known biomarkers that directly associated with liver metabolism or atherosclerosis. Many novel key genes may be hidden in the list of DEGs that associated with atherosclerosis via liver metabolism. Thus, the DEGs can provide rich resource for interpreting and comparing the molecular mechanisms of treatment with different AMPK activators.

### Screening of significant biomedical pathways among DEGs

**Figure 4:**
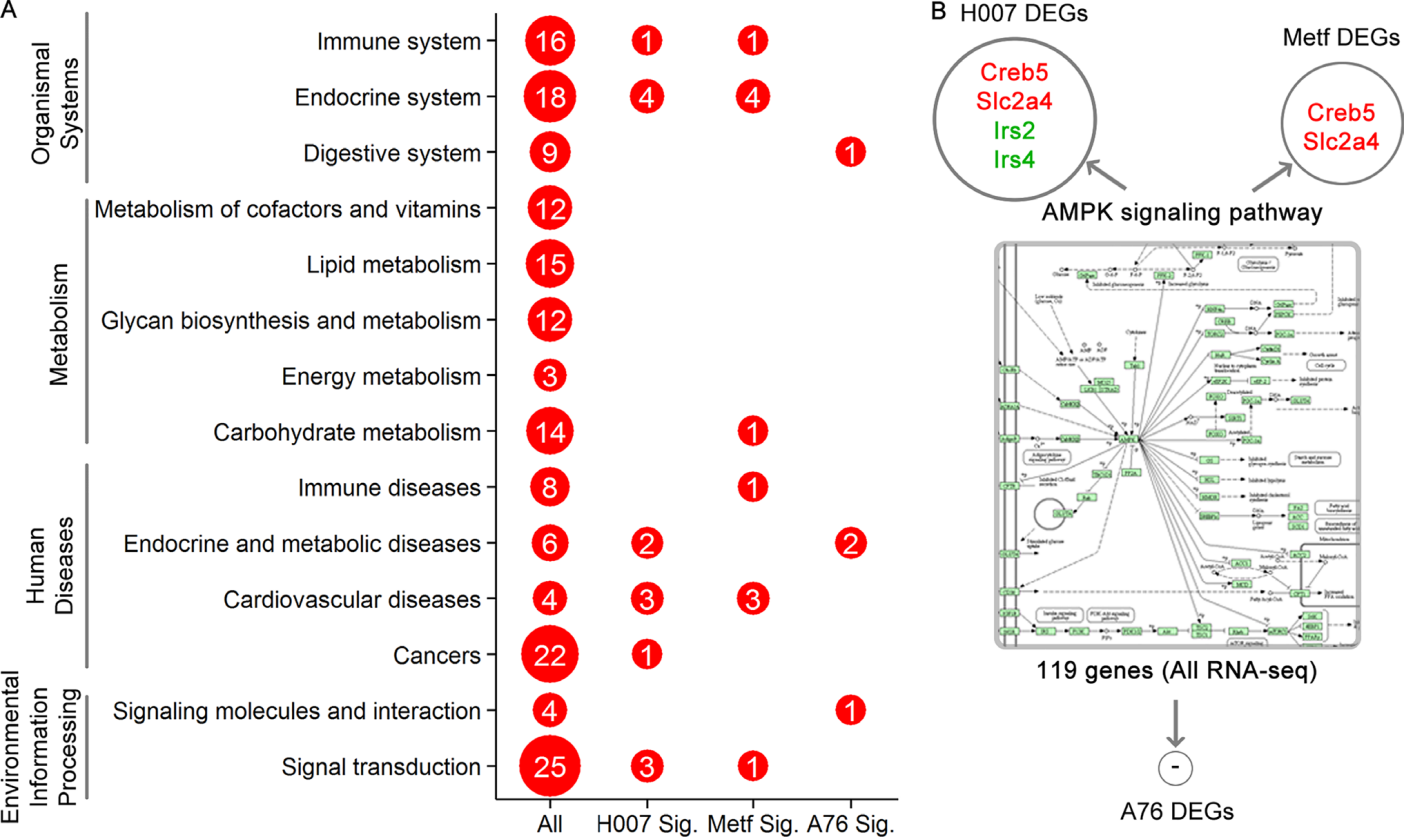
Summary of biomedical pathway analysis (**A**) The numbers of the total pathways and enriched pathways in three groups for each pathway class. (**B**) DEGs in AMPK signalling pathway.

After treating the mice with high-fat food and AMPK activators, we systematically annotated potential biomedical pathways that could be associated with atherosclerosis based on KEGG database (Supplementary Information 4). Liver is the target organ for drug metabolism and lipid metabolism. Thus, various pathways could be involved in the pathogenesis or treatment of atherosclerosis via liver functions. We classified these biomedical pathways into 14 classes, including environmental Information processing (signal transduction and signaling molecules and interaction), diseases (cancers, cardiovascular diseases, endocrine and metabolic diseases, and immune diseases), metabolism (carbohydrate metabolism, energy metabolism, glycan biosynthesis and metabolism, lipid metabolism, and metabolism of cofactors and vitamins), and organismal systems (digestive system, endocrine system, and immune system). In summary, a total of 168 KEGG pathways were annotated for the expression of DEGs, the detailed pathway information (including the numbers of the total pathways and enriched pathways in three groups) for each class are shown in Figure 4A. The DEGs of three experimental groups were mainly observed to be enriched in 21 KEGG pathways (Table 1). Consistent with the results of phenotype analysis, seven statistically significant pathways were observed in both H007 and Metf groups (including calcium signaling pathway, dilated cardiomyopathy, hypertrophic cardiomyopathy, arrhythmogenic right ventricular cardiomyopathy, oxytocin signaling pathway, leukocyte trans-endothelial migration, and regulation of lipolysis in adipocytes).

**Table 1:** Summary of statistically enriched biomedical pathways

Name	UP regulated	DOWN regulated	Fold change	*P* value
**H007**				
Insulin resistance	Ppp1r3a;Slc2a4;Trib3;Pygm;Ppargc1b;Creb5	Ppp1r3c;Irs2	5.0	3.50E-04
Hypertrophic cardiomyopathy (HCM)	Ttn;Myl3;Myl2;Cacna1s;Myh7	Itga10	5.2	1.50E-03
Dilated cardiomyopathy	Ttn;Myl3;Myl2;Cacna1s;Myh7	Itga10	4.9	2.00E-03
cGMP-PKG signaling pathway	Cacna1s;Atp2a1;Mylk2;Mylk4;Creb5;Myh7	Irs2;Irs4	3.5	3.20E-03
Calcium signaling pathway	Gnal;Atp2a1;Cacna1s;Ryr1;Mylk2;Mylk4;Camk4	Cacna1e	3.4	3.50E-03
FoxO signaling pathway	Slc2a4;Gadd45a	Irs2;Irs4;Ccnb1;Cdkn1a;Plk1	3.7	4.30E-03
Type II diabetes mellitus	Slc2a4	Irs2;Irs4;Cacna1e	5.9	6.20E-03
Oxytocin signaling pathway	Ryr1;Cacna1s;Camk4;Mylk2;Mylk4	Ptgs2;Cdkn1a	3.2	9.00E-03
Regulation of lipolysis in adipocytes		Irs2;Irs4;Ptgs2;Npy1r	4.9	1.10E-02
Insulin signaling pathway	Ppp1r3a;Pygm;Slc2a4	Irs2;Irs4;Ppp1r3c	2.9	2.00E-02
Leukocyte transendothelial migration	Myl2;Myl7;Mylpf;Actn2;Actn3		3.2	2.40E-02
Arrhythmogenic right ventricular cardiomyopathy (ARVC)	Cacna1s;Actn2;Actn3	Itga10	3.7	2.60E-02
Ovarian steroidogenesis	Cyp17a1	Ptgs2;Hsd17b1	4.6	3.10E-02
Viral carcinogenesis	Creb5;Actn2;Actn3	Cdkn1a;Hist1h2be;Chek1;Cdc20	2.4	3.20E-02
**Metf**				
Calcium signaling pathway	Htr7;Adcy1;Atp2a1;Cacna1s;Ryr1;Slc25a4;Tnnc2;Phkg1;Mylk2;Mylk4;Camk2a;Camk4;Nos1;Nos2	Cacna1e;P2rx3;Grin2c	4.5	1.00E-06
Dilated cardiomyopathy	Itga2;Sgca;Des;Ttn;Tpm2;Myl3;Myl2;Adcy1;Cacna1s;Cacnb4;Cacng6;Myh7		6.1	2.30E-06
Hypertrophic cardiomyopathy (HCM)	Itga2;Sgca;Des;Ttn;Tpm2;Myl3;Myl2;Cacna1s;Cacnb4;Cacng6;Myh7		6.0	7.40E-06
Arrhythmogenic right ventricular cardiomyopathy (ARVC)	Itga2;Sgca;Des;Cacna1s;Cacnb4;Cacng6;Actn2;Actn3		4.7	5.90E-04
Oxytocin signaling pathway	Ryr1;Cacna1s;Cacnb4;Cacng6;Camk2a;Camk4;Mylk2;Mylk4;Adcy1	Ptgs2	2.8	4.50E-03
Glycolysis / Gluconeogenesis	Pgam2;Eno3;Ldhb;Aldh3b2;Acss1		3.6	1.60E-02
Glucagon signaling pathway	Creb5;Camk2a;Phkg1;Pygm;Pgam2;Ldhb		2.7	3.10E-02
Aldosterone synthesis and secretion	Creb5;Cacna1s;Camk2a;Camk4;Adcy1		2.7	4.30E-02
Primary immunodeficiency	Cd79a;Tnfrsf13c;Cd19		4.0	4.60E-02
Leukocyte transendothelial migration	Cldn8;Myl2;Myl7;Mylpf;Actn2;Actn3		2.4	4.70E-02
Regulation of lipolysis in adipocytes	Adcy1;Plin1	Ptgs2;Npy1r	3.1	4.90E-02
**A76**				
Protein digestion and absorption	Cpa3;Col4a6;Col4a5;Col5a2;Col6a2		4.1	9.20E-03
ECM-receptor interaction	Col4a6;Col4a5;Col6a2;Thbs4;Sv2a		3.8	1.20E-02
Insulin resistance	Trib3;Ppargc1b;Gfpt2	Ppp1r3c;Ppp1r3b	2.9	3.60E-02
Maturity onset diabetes of the young		Onecut1;Bhlha15	6.7	4.20E-02

All the three drugs were AMPK activators, though AMPK signaling pathway is not identified as a significant pathway. There were only 4 and 2 DEGs observed in AMPK signaling pathways for H007 and Metf group respectively (Figure 4B). Since AMPK signaling pathway is highly associated with kinase activities and phosphorylation levels, it was impossible for RNA-seq analysis to discover post-translational or protein changes. Therefore, our study was aimed to identify DEGs that are responsible for atherosclerosis at the level of transcripts. It should be noted that pathway enrichment analysis may also miss some important information for interpreting the mechanism which could be solved by proteomics analysis in the future. For example, although lipid metabolism pathways were not identified as statistically significant, 15 DEGs were distributed in five lipid metabolism pathways (including steroid biosynthesis, arachidonic acid metabolism, steroid hormone biosynthesis, fatty acid elongation, glycerophospholipid metabolism and glycerolipid metabolism).

**Figure 5:**
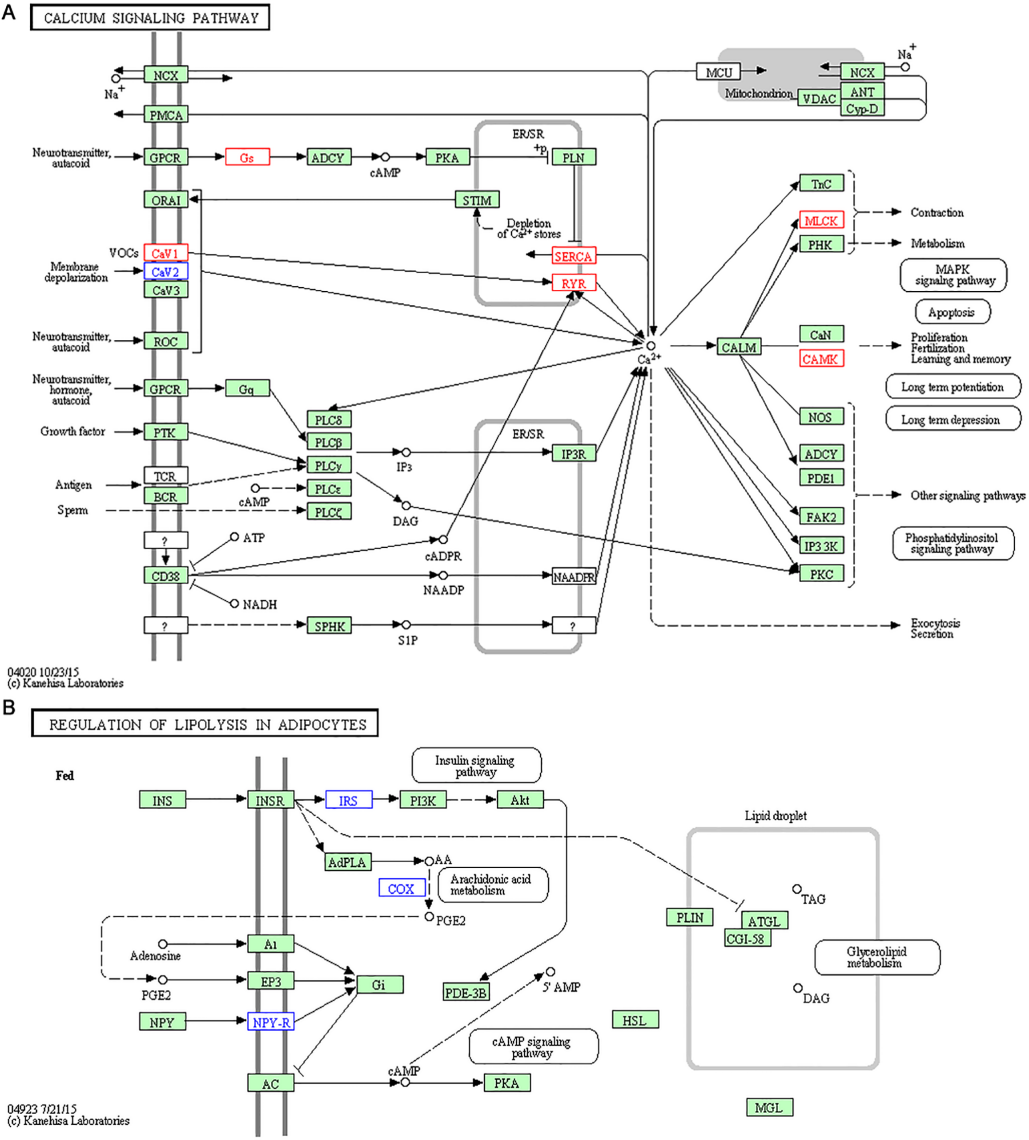
Representative enriched pathways in H007 group (**A**) Calcium signalling pathway. (**B**) Regulation of lipolysis in adipocytes. Genes with red border are up-regulated, while genes with blue border are down-regulated.

In the present study, we mainly focused on the statistically enriched pathways since more DEGs are involved. Among these pathways, some pathways are known to possess important roles in the pathogenesis of atherosclerosis. For example, calcium signaling pathway is enriched in both Metf and H007 group (Figure 5A), microarray analysis of gene expression revealed that mouse aorta also enriched this pathway [18]. Following experiments further verified that calcium signaling pathway is the major controlling factor underlying atherosclerotic lesion development and involved in the inflammatory process of atherogenesis [19, 20]. Although KEGG does not contain pathway that directly associated with atherogenesis, many other diseases could provide indirect pathways and molecular biomarkers for explaining the treatments. There are three cardiovascular diseases associated pathways (including dilated cardiomyopathy, hypertrophic cardiomyopathy, and arrhythmogenic right ventricular cardiomyopathy) enriched in both H007 and Metf group. This enriched pathway could also be a resource for discovering and comparing novel molecular mechanisms. For example, only three down-regulated genes were found in the pathway of regulation of lipolysis in adipocytes for H007 group (Figure 5B). However, two up-regulated and two down-regulated genes were found for Metf group. Previous studies showed that the suppression of lipolysis in adipocytes can activate the AMPK pathway [20], thus regulation of lipolysis in adipocytes could be a new target for atherogenesis. Besides gene difference in the same pathway, there also exists interesting pathway difference between different groups. For example, compared to other groups, many pathways associated with diabetes such as insulin resistance, type II diabetes mellitus and insulin signaling pathway are enriched in H007 group. Diabetes could cause atherosclerosis, and thus may share some common mechanism in pathogenesis [21]. Only H007 group enriched diabetes associated pathways, which indicates that H007 may also be applied in diabetes for treatment via the common pathways.

## DISCUSSION

Recently AMPK activators were observed to have important roles in maintaining the balance between anabolic and catabolic programs for cellular homeostasis in response to metabolic stress. AMPK, as a heterotrimer consisting of a catalytic α subunit and regulatory β and γ subunits, is considered to be a major therapeutic target for the treatment of metabolic diseases including type 2 diabetes, obesity and atherosclerosis [6]. The anti-diabetic drug metformin activates AMPK indirectly. It is a derivative of guanidine acts by promoting insulin-stimulated glucose uptake in muscle and lowers hepatic glucose output, it also affects lipid metabolism, lowering plasma triglycerides and free fatty acids, the latter possibly due to inhibition of catecholamine-stimulated lipolysis [22–24]. A-769662 can promote whole-body fat oxidation in mice acts by directly binding on the AMPKβ1 subunits [12], while our previous work showed that triacetyl-3-hydroxyphenyladenosine (IMM-H007) activates AMPK by directly binding to the γ subunit [10]. Although all of these activities have atheroprotective effects, the importance of AMPK and the mechanism of AMPK activation for atheroprotection are not fully understood. Thus, in this study we chose three different AMPK activators (IMM-H007, A-769662 and Metformin) to compare the effects and discover the molecular mechanism of the treatment of atherosclerosis using these activators.

From the graphical representation of lesion area in the aortic root of control group and drugs-treated apoE^–/–^ mice, with maximum of 353.8682 (units) for control group and 202.6138 (units), 193.4254 (units) and 214.2085 (units) for IMM-H007, Metformin and A-769662 respectively. And from graphical representation of CD68 positive area in cryosection of aorta determined by software ImageJ analysis revealed, that 27.7% positive area for the control group and 14.2%,17.6% and17.5% for IMM-H007, Metformin and A-769662 respectively.

Our results suggested that all three AMPK activators were found to have good effect in treating atherosclerosis, since comparing with the control group the AMPK treated group was able to demonstrate noticeable difference in the lesion areas, in both whole aorta and cryosections staining. We were successfully able to develop the disease model and mimic the therapeutic effect and identify the known atherosclerosis related markers through the transcriptional group. Our study had limitations in many aspects including the transcription levels of protein and metabolic group, wherefore more elaborated studies should be conducted in the future to provide new clues for developing novel drugs which may provide new markers and mechanisms to access and treat atherosclerosis.

## MATERIALS AND METHODS

### Animal model

In this experiment we used Male apoE^–/–^ mice on the C57BL/6 background, weighing between 20–25 g. All mice were purchased from Vital River Laboratory Animal Technology Co. Ltd. (Beijing, China). Mice were fed with high fat diet (10% lard, 1.2% cholesterol) in a pathogen-free animal environment with 12 h light/12 h dark cycles under controlled temperature. They were treated with CMC-Na as the control group, A-769662 (30 mg/kg, i.p.), Metformin (260 mg/kg, i.g.), and IMM-H007 (200 mg/kg, i.g.) once daily. We recorded the weight and food intake every week. All procedures were performed in accordance with the regulations of the Institutional Animal Care and Use Committee of the Institute of Materia Medica, Chinese Academy of Medical Sciences and Peking Union Medical College (Beijing, China).

### Evaluation of aortic atherosclerotic lesion formation

To assess the differences in atherosclerotic lesions size between the experimental and control groups, en face lesion analysis was carried out after 10 weeks treatment with high fat diet (HFD) or AMPK activators, the whole aorta and 7 μm-thick frozen section of aortic sinus were obtained and stained with Oil Red O. The mice aorta preparation and the quantification of atherosclerosis lesions were conducted as previously described [25]. Lesion areas were marked using ImageJ software.

### Atherosclerosis analysis

To induce atherosclerosis in apoE^–/–^ mice, they were fed with western diet (containing 1.25% cholesterol) and libitum for 10 weeks. Mice aorta preparation and atherosclerosis quantification were conducted with modifications as previously described [25]. The entire aorta was stained with Oil Red O for En face analysis and the images were captured with a Nikon D600 digital camera. Aortic roots were frozen in OCT, and 7-μm-thick sections were stained with Oil Red O for lipid content and anti-CD68 antibody for macrophage analysis. Lesion areas were quantified with ImageJ software.

### Western blot analysis

The proteins in apoE^–/–^ liver were lysed in RIPA buffer containing a cocktail of protease and phosphatase inhibitors (Roche). After quantified with BCA protein assay kit (MACGENE, China), proteins were electrophoresed by SDS-PAGE on 10% gels and transferred to PVDF membrane (Millipore). The 5% BSA was used to block PVDF membranes. Then membranes were incubated with antibodies antibodies aganist p-AMPK, AMPK, β-actin (all from Cell Signaling Technology). Membranes were subsequently incubated with HRP-conjugated goat anti-rabbit secondary antibodies (ZSGB-BIO, China) and SuperSignal West Pico chemiluminescent substrate (Millipore).

### Illumina RNA-Seq and data analysis

The entire RNA was treated with RQ1 DNase (promega) to remove DNA. The quality and quantity of the purified RNA were determined by measuring the absorbance at 260 nm/280 nm (A260/A280) using smartspec plus (BioRad). RNA integrity was further verified by 1.5% Agarose gel electrophoresis. Then for each sample, 10 μg of total RNA was used for RNA-seq library preparation. Polyadenylated mRNAs were purified and concentrated with oligo (dT)-conjugated magnetic beads (invitrogen) before they were used for directional RNA-seq library preparation. Purified mRNAs were iron fragmented at 95°C followed by end repair and 5′ adaptor ligation. Then reverse transcription was performed with RT primer harboring 3′ adaptor sequence and randomized hexamer. The cDNAs were purified and amplified and the PCR products corresponding to 200–500 bps were also purified and quantified and were stored at –80°C until used for sequencing. For high-throughput sequencing, the libraries were prepared following the manufacturer's instructions and applied to illumina NextSeq 500 system for 151 nt pair-end sequencing by ABlife. Inc. (Wuhan, China). Following analysis of raw data (Fastq files) was obtained by ABlife Inc. using a similar workflow as previously described [26]. The accurate readings were aligned in the Ensembl Mus musculus genome using TopHat2 software [27]. Then, gene expression levels were calculated using FPKM (reads per kilobase per million mapped reads) method, which considered the length and read counts uniquely mapped to each gene. The edgeR software was used to further identify DEGs between experimental and control groups [28]. Genes with *P* value less than 0.01 and fold change larger than 2 were considered statistically significant.

### Statistical analysis

Correlation analysis of experimental replications and groups were performed by the R soft, while clustering analysis was performed by the MeV toolkit [29]. For both correlation and clustering analysis, Pearson method was applied. The known phenotype information were obtained from the MGI (Mouse Genome Informatics) database [30]. The gene-phenotype relation network was constructed using the Cytoscape software (Version: 3.2) [31]. Biomedical pathways were based on the KEGG (Kyoto Encyclopedia of Genes and Genomes) database [32]. For enrichment statistics, the whole genes identified in the RNA-seq analysis was set as background. Pathways with *P* value (by Fisher's exact test) less than 0.05 and fold change larger than 2 were considered statistically significant.

## SUPPLEMENTARY MATERIALS










