# Supplementary material for: Comparative transcriptomic analysis of mice liver treated with different AMPK activators in a mice model of atherosclerosis
Source: Oncotarget. 2017 Feb 2;8(10):16594–604. doi: 10.18632/oncotarget.15027 (PMC5369987; doi:10.18632/oncotarget.15027)
Supplement: Supplementary file 1 [file oncotarget-08-16594-s001.pdf]

Comparative transcriptomic analysis of mice liver treated with different AMPK activators in a mice model of atherosclerosis

Supplementary Materials

Supplementary Information 1: A total of 20047 unique genes were identified in mouse liver treated with different AMPK activators. See Supplementary_Information_1

Supplementary Information 2: A total of 799 DEGs were identified in treatment groups compared to model group. See Supplementary_Information_2

Supplementary Information 3: A total of 46 DEGs were mapped to 49 subcalsses of phenotypes. See Supplementary_Information_3

Supplementary Information 4: Annotated potential biomedical pathways that could be associated with atherosclerosis based on KEGG database. See Supplementary_Information_4
